# Evaluation of in-vivo anti-*Salmonella* activity of *Uvaria chamae, Lantana camara* and *Phyllantus amarus* used in Benin, West Africa

**DOI:** 10.1186/s12917-020-2266-1

**Published:** 2020-02-10

**Authors:** Boris Legba, Victorien Dougnon, Yossounon Chabi, Carène Gbaguidi, Alidah Aniambossou, Esther Deguenon, Jacques Dougnon, Marc Kpodekon, Lamine Baba-Moussa

**Affiliations:** 1 Research Unit in Applied Microbiology and Pharmacology of natural substances, Research Laboratory in Applied Biology, Polytechnic School of Abomey-Calavi, University of Abomey-Calavi, Cotonou, Benin; 2 Laboratory of Biology and Molecular Typing in Microbiology, Faculty of Science and Technology, University of Abomey-Calavi, Cotonou, Benin; 3 Department of Drugs, Pharmacies and Diagnostic Exploration, Ministry of Health, Cotonou, Benin; 4 Beninese Center for Scientific Research and Innovation, Ministry of Higher Education and Scientific Research, Cotonou, Benin

**Keywords:** *Salmonella* Typhimurium ATCC 14028, Public health, Salmonellosis, *Uvaria chamae*, Improved traditional Medicine

## Abstract

**Background:**

*Uvaria chamae (Annonaceae), Phyllantus amarus (Phyllantaceae)* and *Lantana camara (Verbenaceae)* are empirically alleged to be used as Beninese medicinal plants in the treatment of salmonellosis. This study aimed to produce scientific data on in vitro and in vivo *efficacy* of *Uvaria chamae, Lantana camara* and *Phyllantus amarus* on multiresistant *Salmonella spp* isolated in Benin.

**Results:**

After performing in vitro tests on aqueous and ethanolic extracts of these plants, only the aqueous extract of *Uvaria chamae* (leaves) showed the best anti-*Salmonella*’s activity and was used for this in vivo experiment. The induction of salmonellosis revealed 9 × 10^8^ CFU/ml was the optimal concentration triggering and maintaining symptoms in chicks. This infective concentration was used for in vivo assessment. Twenty-four hours post inoculation, the symptoms of salmonellosis (wet cloaca, diarrhea stools and somnolence) were observed in infected groups*.* After 7 days of treatment, the reduction of bacterial load at 100 mg/L, 200 mg/L, 400 mg/L of the extract was respectively 85%, 52.38% and 98% for *Uvaria chamae, Phyllantus amarus* and *Lantana camara* in the chick’s groups infected with *Salmonella* Typhimurium ATCC 14028. On the other hand, colistin completely cancelled the bacterial load (reduction rate of 100%).

With the groups infected with *Salmonella spp* (virulent strain), the reduction rate of bacterial load at 100 mg/L, 200 mg/L, 400 mg/L of extract was 0%, 98.66%, and 99.33%. The extracts at 200 and 400 mg/L were more active than colistin, which reduced the bacterial load by 33.33%.

The toxicity tests did not show any negative effect of Colistin and the *Uvaria chamae*’s extract on the biochemical and hematological parameters of the chicks.

**Conclusion:**

The aqueous extract of *Uvaria chamae* is active in vitro and in vivo on multiresistant strains of *Salmonella enterica*. This plant is a good candidate for the development of an improved traditional medicine for the management of salmonellosis.

## Background

*Salmonella* spp. is one of the major causes of foodborne illness worldwide. This germ is the source of zoonotic infections in humans and some animals. Transmission to humans can occur from contaminated food of animal origin. Pork, poultry and meat products are often cited [1].

*Salmonella* spp. belongs to the Enterobacteriaceae family. It is a Gram- bacilli and facultative anaerobic bacterium. The genus is divided into three main species including *Salmonella enterica*, *Salmonella bongori* and *Salmonella subterranean* [2]. It is one of four main causes of diarrheal diseases worldwide [3]. There were about 94 million cases of gastroenteritis with 155,000 deaths globally every year [4]. Out of these cases, it is considered that 80.3 million were foodborne origin [5]. In poultry, the epidemiologically important serotypes associated with the majority of human salmonellosis are *Salmonella* Enteritidis, *Salmonella* Typhimurium, *Salmonella* Newport and *Salmonella* Heidelberg [6–9].

The emergence of multiresistance of *Salmonella* strains towards reference antibiotics such as fluoroquinolones and third-generation cephalosporins became a public health problem worldwide [10–13]. As an alternative to antimicrobial resistance, exploration of medicinal plants with anti-*Salmonella* activity is becoming very common in West Africa. In vitro evidence of antibacterial activity of plant extracts on *Salmonella* strains has been demonstrated [14] but there is a few data about in vivo efficacy of medicinal plants against this bacteria.

The difficulty of choosing a suitable study model, the complexity of such research work, and the still limited data on the physiology of *Salmonella* strains could explain this situation. This study aimed to the experimental induction of salmonellosis in chicks as animals’ models. These data can be a good starting point for in vivo efficacy testing of herbal extracts on *Salmonella spp*. Indeed, promoting medicinal plants used in the traditional treatment of salmonellosis involves a structured approach. At this time, an ethnopharmacological survey revealed 57 species of medicinal plants used in the treatment of salmonellosis in Benin [15]. Based on quotation frequency and literary data, *Phyllantus amarus, Senna siamea, Uvaria chamae and Lantana camara* have been selected. Toxicological, chemical and antibacterial (to ten enteropathogens) characterization have been done. This study showed interesting contents in polyphenols and flavonoids and an effective antibacterial activity at 100 mg/mL with Minimal Inhibitory Concentration (MIC) between 100 and 25 mg/mL and inhibition diameters between 7.5 and 21 mm [16].

With these results, it was found necessary to evaluate the in vitro and in vivo efficacy of extracts of *Uvaria chamae*, *Phyllantus amarus* and *Lantana camara* on multiresistant *Salmonella spp* isolated in Benin.

## Results

### In vitro anti-*Salmonella* activity of *Uvaria chamae*, *Phyllantus amarus* and *Lantana camara*

**Fig. 1:**
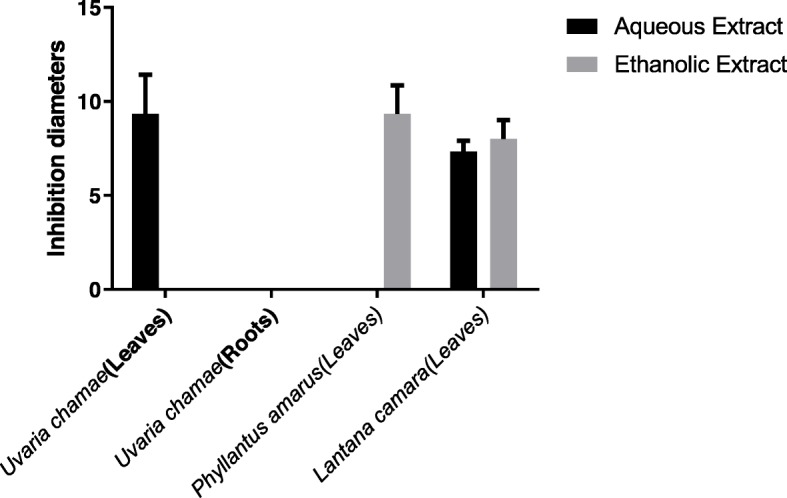
Antibacterial activity of aqueous and ethanolic extracts of Uvaria chamae, Phyllantus amarus and *Lantana camara* on Salmonella Typhimurium ATCC 14028

*Salmonella* Typhimurium ATCC 14028 was sensitive to aqueous extract of *Uvaria chamae* (leaves)*,* ethanolic extract of *Phyllantus amarus* (leaves), ethanolic and aqueous extract of *Lantana camara* (leaves)*.* The aqueous extract of *Uvaria chamae* (leaves) showed the best inhibition diameter (9.33 ± 2.08 mm) (Fig. 1). There is no significant difference between inhibition diameters (*P* ≥ 0.05) of active extracts.

**Fig. 2:**
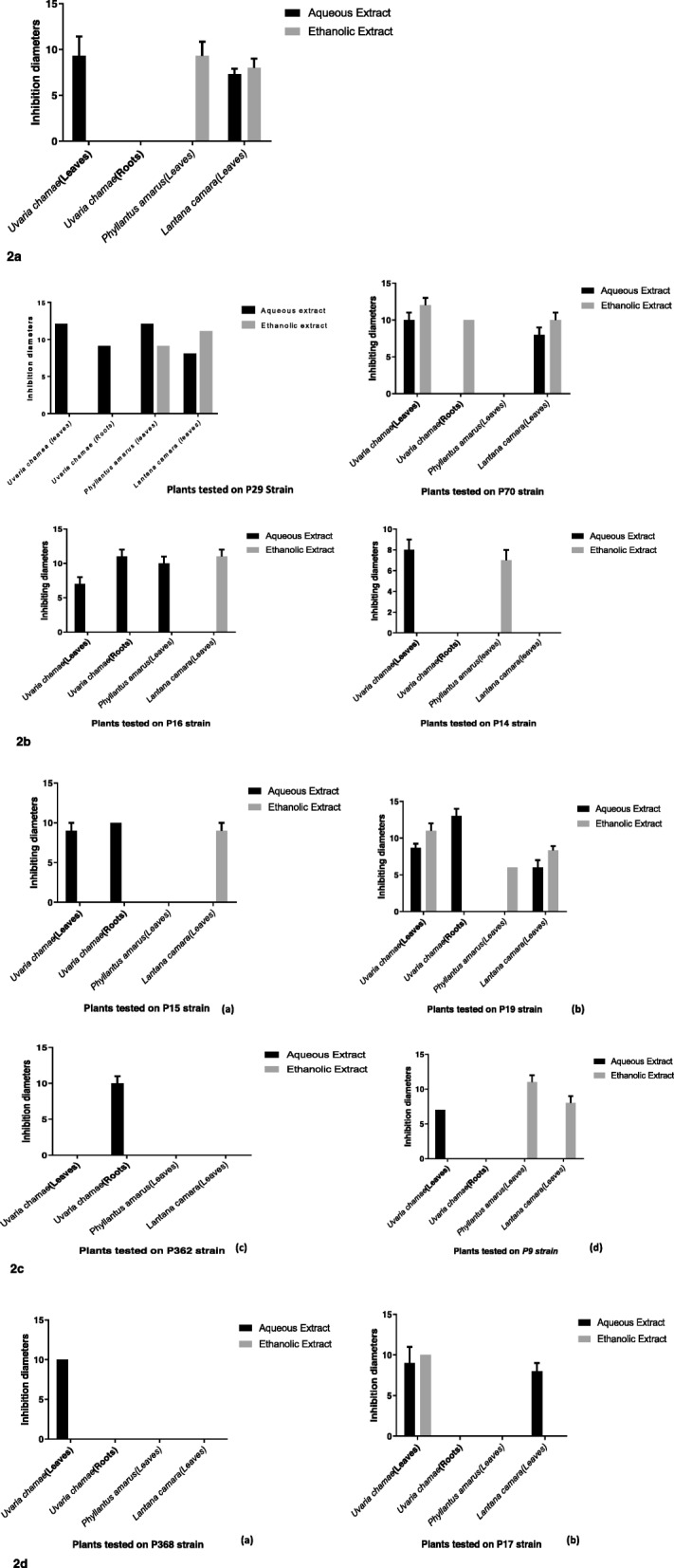
Antibacterial activity of aqueous and ethanolic extracts of *Uvaria chamae*, *Phyllantus amarus* and *Lantana camara* on multiresistant *Salmonella spp*. isolated in Benin

Figure 2 presents antibacterial activity of aqueous and ethanolic extracts of *Uvaria chamae, Phyllantus amarus* and *Lantana camara* on multiresistant strains of *Salmonella spp* isolated in Benin. Out of ethanolic extract (roots) of *Uvaria chamae*, all extracts showed variable susceptibility to multiresistant strains of *Salmonella spp.* Leaves aqueous extract of *Uvaria chamae* was active on 90% of *Salmonella spp*. Root aqueous extract of *Uvaria chamae* showed the best inhibition diameter (13 ± 1 mm on P19 *Salmonella spp).*

The Minimum Inhibitory Concentration (MIC), the Minimum Bactericidal Concentration (MBC) and the antibiotic potency (ap) of the plant extracts studied on the tested strains are summarized in Table 1. MIC range from 1.625 (Leaves ethanolic extract of *Uvaria chamae* on P17 *Salmonella* spp) to 100 mg/ml. Leaves aqueous extract of *Uvaria chamae* was bacteriostatic on *Salmonella* Typhimurium ATCC 14028 and *Salmonella* spp. P19.

**Table 1:** MIC (mg/ml), MBC (mg/ml) and a. p. of the aqueous and Ethanolic extracts of the plants on *Salmonella spp*.

Extracts	Parameters	*P9*	*P70*	*P16*	*P14*	*P15*	*P19*	*P362*	*P368*	*P17*	*P29*	*ST*
*U.Chamae* Leaves aqueous extract	MIC	12.5	25	6.25	6.25	6.25	12.5	–	12.5	3.25	12.5	6.25
	MBC	100	> 100	> 100	> 100	> 100	100	–	50	100	> 100	100
	a.p.	8	–	–	–	–	8	–	4	32	–	8
*U.Chamae* Leaves ethanolic extract	MIC	3.125	–	–	–	–	3.125	–	–	1.5265	–	–
	MBC	> 100	–	–	–	–	> 100	–	–	100	–	–
	a.p.	–	–	–	–	–	–	–	–	65.51	–	–
*U.Chamae* Roots aqueous extract	MIC	–	–	50	–	6.25	–	12.5	–	–	25	–
	MBC	–	–	> 100	–	> 100	–	> 100	–	–	> 100	–
	a.p.	–	–	–	–	–	–	–	–	–	–	–
*U.Chamae* Roots ethanolic extract	MIB	–	–	–	–	–	–	–	–	–	–	–
	MBC	–	–	–	–	–	–	–	–	–	–	–
	a.p.	–	–	–	–	–	–	–	–	–	–	–
*P. amarus* Leaves aqueous extract	MIC	–	–	50	–	–	–	100	–	–	50	–
	MBC	–	–	> 100	–	–	–	> 100	–	–	> 100	–
	a.p.	–	–	–	–	–	–	–	–	–	–	–
*P.amarus* Leaves ethanolic extract	MIC	6.25	–	–	25	–	–	–	–	–	12.5	12.5
	MBC	> 100	–	–	> 100	–	–	–	–	–	> 100	> 100
	a.p.	–	–	–	–	–	–	–	–	–	–	–
*L.camara* Leaves aqueous extract	MIB	–	–	–	–	–	–	–	–	–	–	–
	MBC	–	–	–	–	–	–	–	–	–	–	–
	a.p.	–	–	–	–	–	–	–	–	–	–	–
*L.camara* Leaves ethanolic extract	MIB		6.25			3.125					12.5	12.5
	MBC		> 100			> 100					> 100	> 100
	a.p.	–	–	–	–	–	–	–	–	–	–	–

*ST Salmonella* Typhimurium ATCC 14028

*MIC* Minimum Inhibitory Concentration, *MBC* Minimum Bactericidal Concentration, *a.p* Antibiotic potency

### In vivo anti-*Salmonella* activity of aqueous extracts (leaves) of *Uvaria chamae* using chick models

#### Preliminary test

This step aimed to choose the optimal effective concentration of inoculum for *Salmonella*’s induction. Twenty-four hours after infection, wet cloaca and diarrhea stools were detected in groups 1, 2 and 3 infected with 3 × 10^8^ CFU/ml (Concentration 1), 6 × 10^8^ CFU/ml (Concentration 2), 9 × 10^8^ CFU/ml (Concentration 3) respectively whereas these symptoms were absent in group 4, which received distilled water only. These symptoms were present until the 10^th^ day of observation in the group 3 while they disappeared between the fifth and the 7^th^ day in the group 2 (Table 2). However, no deaths were recorded. *Salmonella spp*. was investigated in faecal samples to support clinical observations. The strains were detected in chicks of group 2 and 3, 3 days after salmonellosis induction. They were present till the ninth day for group 3 whereas they disappeared from feces on the sixth day for group 2 (Table 3). On the ninth day, a bacterial count was made on the faeces samples to assess the bacterial load. The results are shown in Table 4.

**Table 2:** Salmonellosis Symptoms in three-day-old chicks inoculated with S*almonella Typhimurim* ATCC 14028(*n* = 3 for each concentration of inoculum)

Groups	Salmonellosis symptoms	Days Post infection									
		1	2	3	4	5	6	7	8	9	10
1 (*3.0 10*^*8*^*UFC/ml*)	*Wet cloaca*	–	–	–	–	–	–	–	–	–	–
	*diarrheal stool*	+	–	–	–	–	–	–	–	–	–
	*Somnolence*	–	–	–	–	–	–	–	–	–	–
2 (*6.0 10*^*8*^*UFC/ml*)	*Wet cloaca*	+	+	+	+	+	–	–	–	–	–
	*diarrheal stool*	+	+	+	+	+	+	+	–	–	–
	*Somnolence*	–	+	–	–	–	–	–	–	–	–
3 (*9.0 10*^*8*^*UFC/ml*)	*Wet cloaca*	+	+	+	+	+	+	+	+	+	+
	*diarrheal stool*	+	+	+	+	+	+	+	+	+	+
	*Somnolence*	–	–	+	+	–	–	–	–	–	–
4 (Distilled Water)	*Wet cloaca*	–	–	–	–	–	–	–	–	–	–
	*diarrheal stool*	–	–	–	–	–	–	–	–	–	–
	*Somnolence*	–	–	–	–	–	–	–	–	–	–

Legend: Absence (−), Presence (+)

**Table 3:** *Salmonella* detection in fecal samples of three-day-old chicks inoculated with *Salmonella* Typhimurim ATCC 14028 (*n* = 3 for each concentration of inoculum)

Groups	Detection of *Salmonella* at Days Post infection				
	0	3	6	9	
G1 (*3.0 10*^*8*^*UFC/ml*)	–	–	–	–	
G2 (*6.0 10*^*8*^*UFC/ml*)	–	+	–	–	
G3 (*9.0 10*^*8*^*UFC/ml*)	–	+	+	+	
G4 (Distilled Water)	–	–	–	–	

Legend: Absence (−), Presence (+)

**Table 4:** *Salmonella* count in fecal samples of three-day-old chicks inoculated with *Salmonella* Typhimurim ATCC 14028

Groups	Bacterial load at Day 9 (CFU/g)
G1 (*3.0 10*^*8*^*UFC/ml*)	0
G2 (*6.0 10*^*8*^*UFC/ml*)	0
G3 (*9.0 10*^*8*^*UFC/ml*)	1.67. 10^3^
G4(Distilled Water)	0

Clinical observations suggest that only 9 × 10^8^ UFC/ml could trigger and maintain the symptoms of salmonellosis.

### In vivo efficacy of leaves aqueous extract of *U. chamae* on *Salmonella* Typhimurium ATCC 14028 (reference strain) and *Salmonella spp*. P19 (virulent strain)

Twenty-four hours after induction, symptoms were detected in all infected chicks. Diarrheal stools were abundant. The cloacae of the chicks were wet and somnolence was noticed in some chicks (Tables 5, 6, 7, 8, 9 and 10). During the 9 days of monitoring, including 7 days of treatment, these symptoms evolved considerably according to the groups. They remained persistent in infected and untreated chicks (group 2). However, diarrhea was slightly reduced at the last days of monitoring, indicating a progression of the disease to asymptomatic carriage (Tables 7 and 8).

**Table 5:** Salmonellosis Symptoms in three-day-old chicks inoculated with S*almonella* Typhimurim ATCC 14028 and treated with Leaves aqueous extract of *U. Chamae* and Colistin (n = 18 for each group)

Groups	Salmonellosis symptoms	Days Post infection								
		1	2	3	4	5	6	7	8	9
G1 (*non-infected and non-treated*)	*Wet cloaca*	–	–	–	–	–	–	–	–	–
	*diarrheal stool*	–	–	–	–	–	–	–	–	–
	*Somnolence*	–	–	–	–	–	–	–	–	–
G2 *(infected and untreated)*	*Wet cloaca*	+	+	+	+	+	+	+	+	+
	*diarrheal stool*	+	+	+	+	+	+	+	+	+
	*Somnolence*	–	+	+	+	+	+	–	–	–
G3 *(Infected and treated with 200 mg/l of Colistin)*	*Wet cloaca*	+	+	+	+	+	–	–	–	–
	*diarrheal stool*	+	+	+	+	+	+	–	–	–
	*Somnolence*	+	+	+	–	–	–	–	–	–
G4 *(infected and treated with 100 mg/l of Uvaria chamae leaves aqueous extract)*	*Wet cloaca*	+	+	+	+	+	+	–	–	–
	*diarrheal stool*	+	+	+	+	+	+	+	+	–
	*Somnolence*	+	+	+	–	–	–	–	–	–
G5 *(infected and treated with 200 mg/l of Uvaria chamae leaves aqueous extract)*	*Wet cloaca*	+	+	+	+	+	+	+	–	–
	*diarrheal stool*	+	+	+	+	+	+	–	–	–
	*Somnolence*	+	+	–	–	–	–	–	–	–
G6 *(infected and treated with 400 mg/l of Uvaria chamae leaves aqueous extract)*	*Wet cloaca*	+	+	+	+	+	+	–	–	–
	*diarrheal stool*	+	+	+	+	+	+	+	–	–
	*Somnolence*	+	+	+	+	–	–	–	–	–

**Table 6:** Salmonellosis Symptoms in three-day-old chicks inoculated with P19 S*almonella spp* strain and treated with Leaves aqueous extract of *U. Chamae* and Colistin (*n* = 18 for each group)

Groups	Salmonellosis symptoms	Days Post infection								
		1	2	3	4	5	6	7	8	9
G1 (*non-infected and non-treated*)	*Wet cloaca*	–	–	–	–	–	–	–	–	–
	*diarrheal stool*	–	–	–	–	–	–	–	–	–
	*Somnolence*	–	–	–	–	–	–	–	–	–
G2 *(infected and untreated)*	*Wet cloaca*	+	+	+	+	+	+	+	+	+
	*diarrheal stool*	+	+	+	+	+	+	+	+	+
	*Somnolence*	+	+	+	+	–	–	–	–	–
G3 *(Infected and treated with 200 mg/l of Colistin)*	*Wet cloaca*	+	+	+	+	+	+	–	–	–
	*diarrheal stool*	+	+	+	+	+	+	–	–	–
	*Somnolence*	+	+	+	+	–	–	–	–	–
G4 *(infected and treated with 100 mg/l of Uvaria chamae leaves aqueous extract)*	*Wet cloaca*	+	+	+	+	+	+	+	–	–
	*diarrheal stool*	+	+	+	+	+	+	+	+	+
	*Somnolence*	+	+	+	–	–	–	–	–	–
G5 *(infected and treated with 200 mg/l of Uvaria chamae leaves aqueous extract)*	*Wet cloaca*	+	+	+	+	–	–	–	–	–
	*diarrheal stool*	+	+	+	+	+	+	–	–	–
	*Somnolence*	+	+	+	–	–	–	–	–	–
G6 *(infected and treated with 400 mg/l of Uvaria chamae leaves aqueous extract)*	*Wet cloaca*	3	–	–	–	–	–	–	–	–
	*diarrheal stool*	+	+	+	+	+	+	–	–	–
	*Somnolence*	+	+	–	–	–	–	–	–	–

Legend: Absence (−), Presence (+)

**Table 7:**
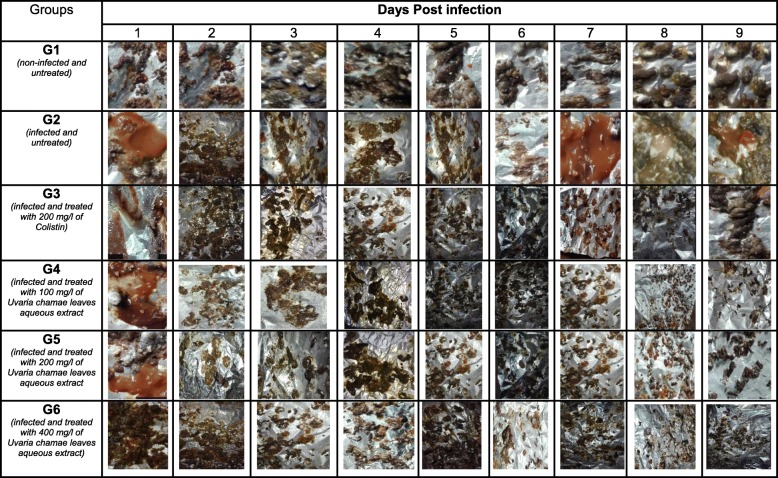
Evolution of faeces aspect from three-day-old chicks inoculated with *Salmonella* Typhimurim ATCC 14028 and treated with Leaves aqueous extract of *U. chamae* and Colistin

**Table 8:**
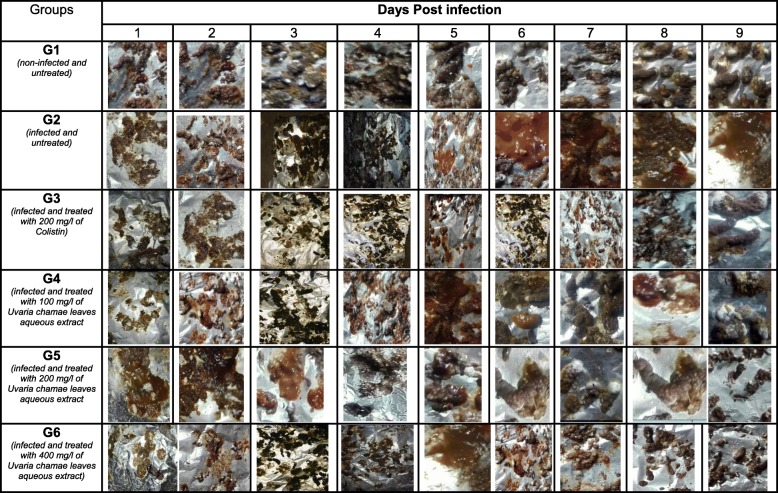
Evolution of faeces aspect from three-day-old chicks inoculated with P19 *Salmonella* strain and treated with Leaves aqueous extract of *U. chamae* and Colistin

**Table 9:**
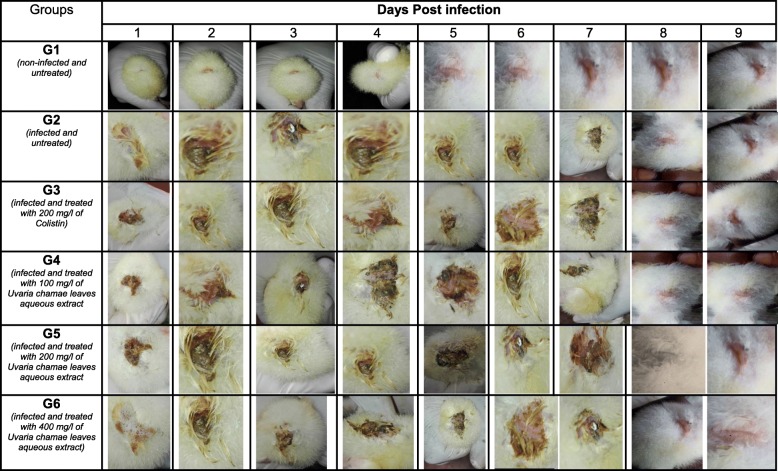
Evolution of cloacal aspect from three-day-old chicks inoculated with Salmonella Typhimurium and treated with Leaves aqueous extract of *U. chamae *and Colistin

**Table 10:**
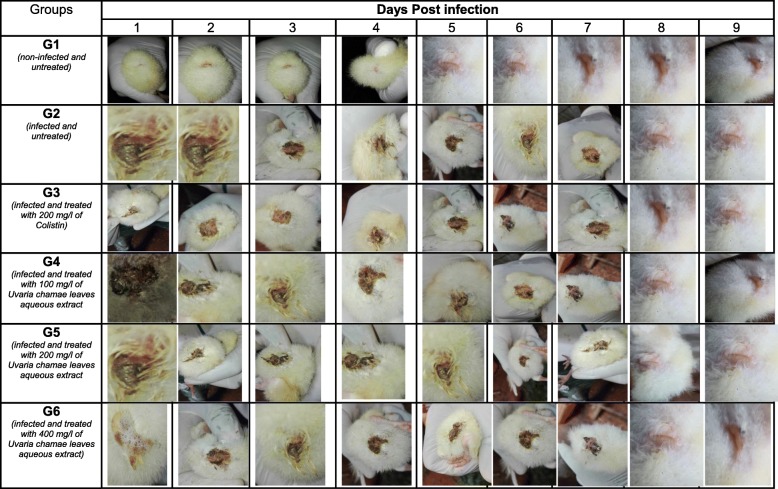
Evolution of cloacal aspect from three-day-old chicks inoculated with P19 *Salmonella* and treated with Leaves aqueous extract of *U. Chamae* and Colistin

**Fig. 3:**
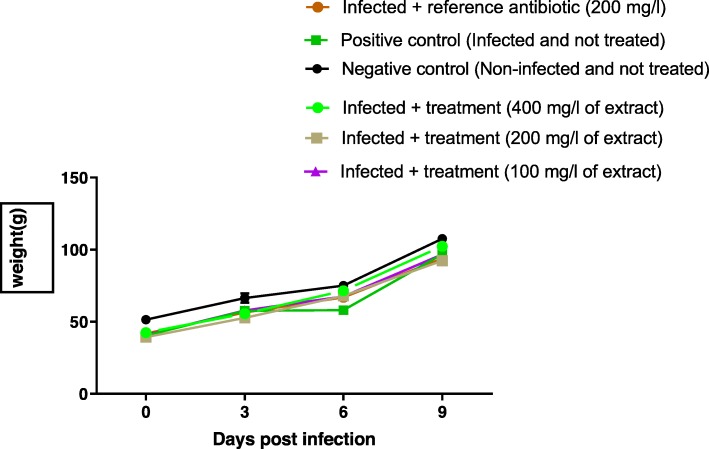
weight changes of chicks orally infected with *Salmonella* Typhimurium 14028 and treated with leaves aqueous extract of *U. chamae* and Colistin

**Fig. 4:**
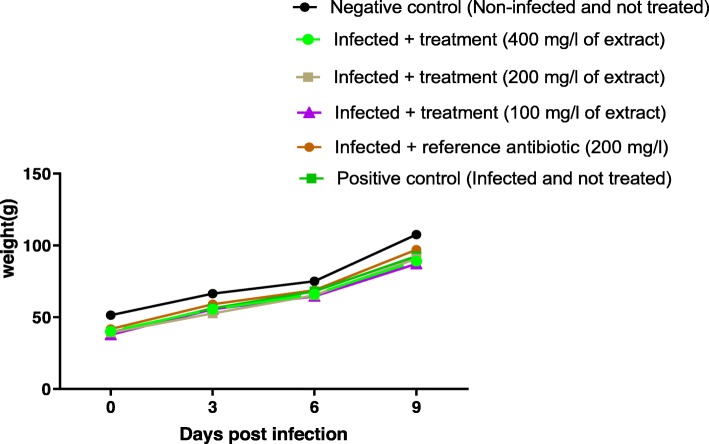
Weight changes of chicks orally infected with *Salmonella spp*. (P19) and treated with leaves aqueous extract of *U. chamae* and Colistin

Throughout the monitoring, there was a progressive increase in the weight of the chicks of all the groups (Figs. 3 and 4). For chicks infected by *Salmonella* Typhimurium ATCC 14028, there was no significant difference in weight change compared to other groups (*p* ≥ 0.05) (Fig. 3). Same observation was done in chicks infected with *Salmonella* spp. P19 (virulent strain) (*p* ≥ 0.05) (Fig. 4).

**Fig. 5:**
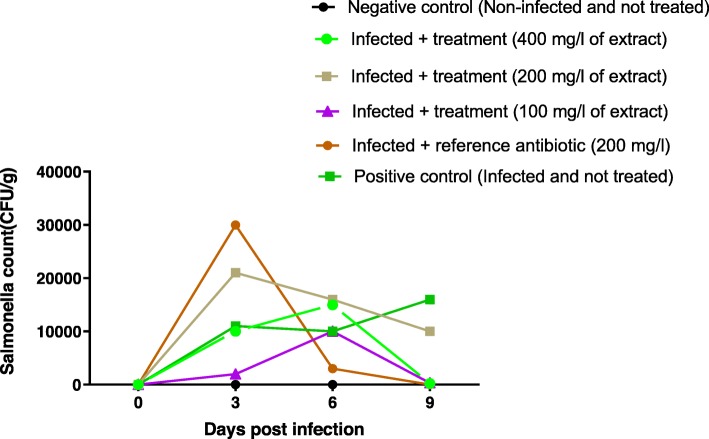
Enumeration of *Salmonella* from feces of chicks orally infected with *S.* Typhimurium 14028 and treated with leaves aqueous extract of *U.chamae* and Colistin

Three days after infection with *Salmonella* Typhimurium, the bacterial load of *Salmonella* increased in all infected chicks, but differentially according to the groups. The bacterial load at day 3 was in the range 2000–30,000 CFU/g. Treatment of the chicks with the extracts and colistin started at day 3 and continued until day 9. During treatment, the bacterial load gradually decreased in group 3 treated with colistin and disappeared on Day 9. In group 2 (infected and untreated), the bacterial load decreased slightly between the third and sixth day (11,000 to 10,000 CFU/g) before undergoing a considerable increase on the ninth day (10,000 to 16,000 CFU/g). In groups 4, 6 respectively treated with 100 and 400 mg/l of the extract, the bacterial load was first increased before falling down on day 9. In group 5 (treated with the extract at 200 mg/l), a gradual decline from the 3rd to the 9th day (21,000 to 10.000 CFU/g) (Fig. 5) was observed. In group 1 (unfected chicks), the bacterial load remained zero.

**Fig. 6:**
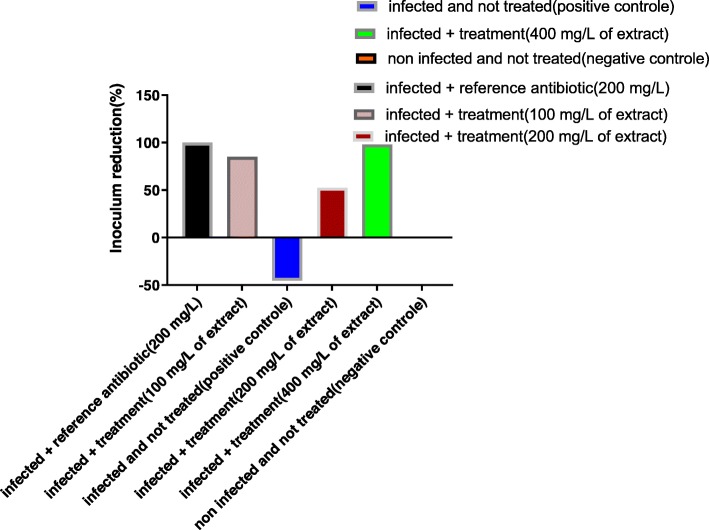
Inoculum reduction of *Salmonella* Typhimurium 14028 between Day 3 and Day 9(Seven-day treatment)

Bacterial load reduction between the 3rd and the 9th day after the infection (7 days of treatment) was assessed globally and summarized in the Fig. 6. The bacterial load increased by 45.45% in group 2 (infected and untreated). It was reduced by 100% in lot 3 (treated with colistin). Treatment with leaves aqueous extract of *Uvaria chamae* also showed remarkable efficacy (bacterial load reduction between 52.38 and 98%).

**Fig. 7:**
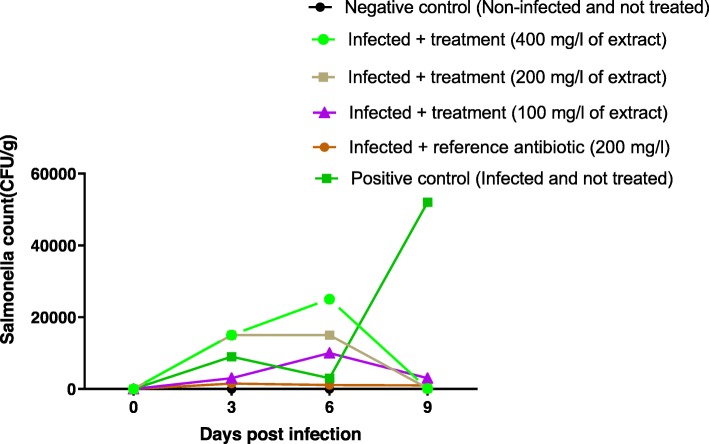
Enumeration of *Salmonella* from faeces of chicks orally infected with *Salmonella spp*. (P19) and treated with leaves aqueous extract of *U. chamae* and Colistin

Three days after infection with *Salmonella* spp. (virulent strain) (Fig. 7), the bacterial load of *Salmonella* increased in all infected chicks. The bacterial load at day 3 was in the range 3000–15,000 CFU/g. Treatment of the chicks with the extracts and colistin started on day 3 and continued until day 9. During treatment, the bacterial load gradually decreased from 1500 to 1000 CFU/g in group 3 treated with colistin. In group 2 (infected and untreated), the bacterial load decreased slightly between the third and sixth day (9000 to 3000 CFU/g) before undergoing a considerable increase on the ninth day (3000 to 52,000 CFU/g). In groups 4, 6 respectively treated with 100 and 400 mg/l, the bacterial load was first increased before falling on day 9, whereas in group 5 treated with the extract at 200 mg/ l, bacterial load was constant to 15,000 CFU/g between day 3 and day 6 and decreased to 200 CFU/g at day 9. In group 1 (non-infected chicks), the bacterial load remained zero.

**Fig. 8:**
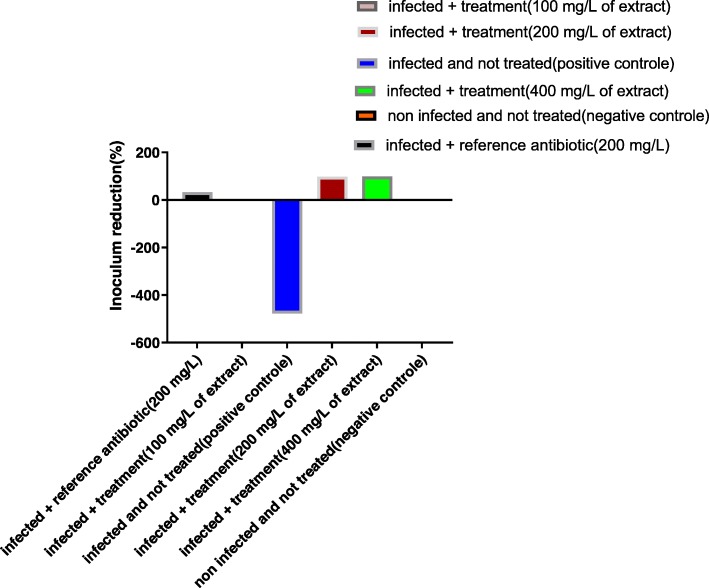
Inoculum reduction of *Salmonella spp*. (P19) between Day 3 and Day 9 (Seven-day treatment)

Bacterial load reduction between the 3rd and the 9th day after the infection (7 days of treatment) was assessed globally and summarized in the Fig. 8. The bacterial load increased by 477.77% (4.7 times) in group 2 (infected and untreated). It was reduced by 33.33% in lot 3 (treated with Colistin), 98.66% in group 5, 99.33% in group 6. The extract at 100 mg/l did not allow a reduction of the bacterial load (group 4).

### Effects of leaves aqueous extract of *Uvaria chamae* on biochemical and hematological parameters

**Fig. 9:**
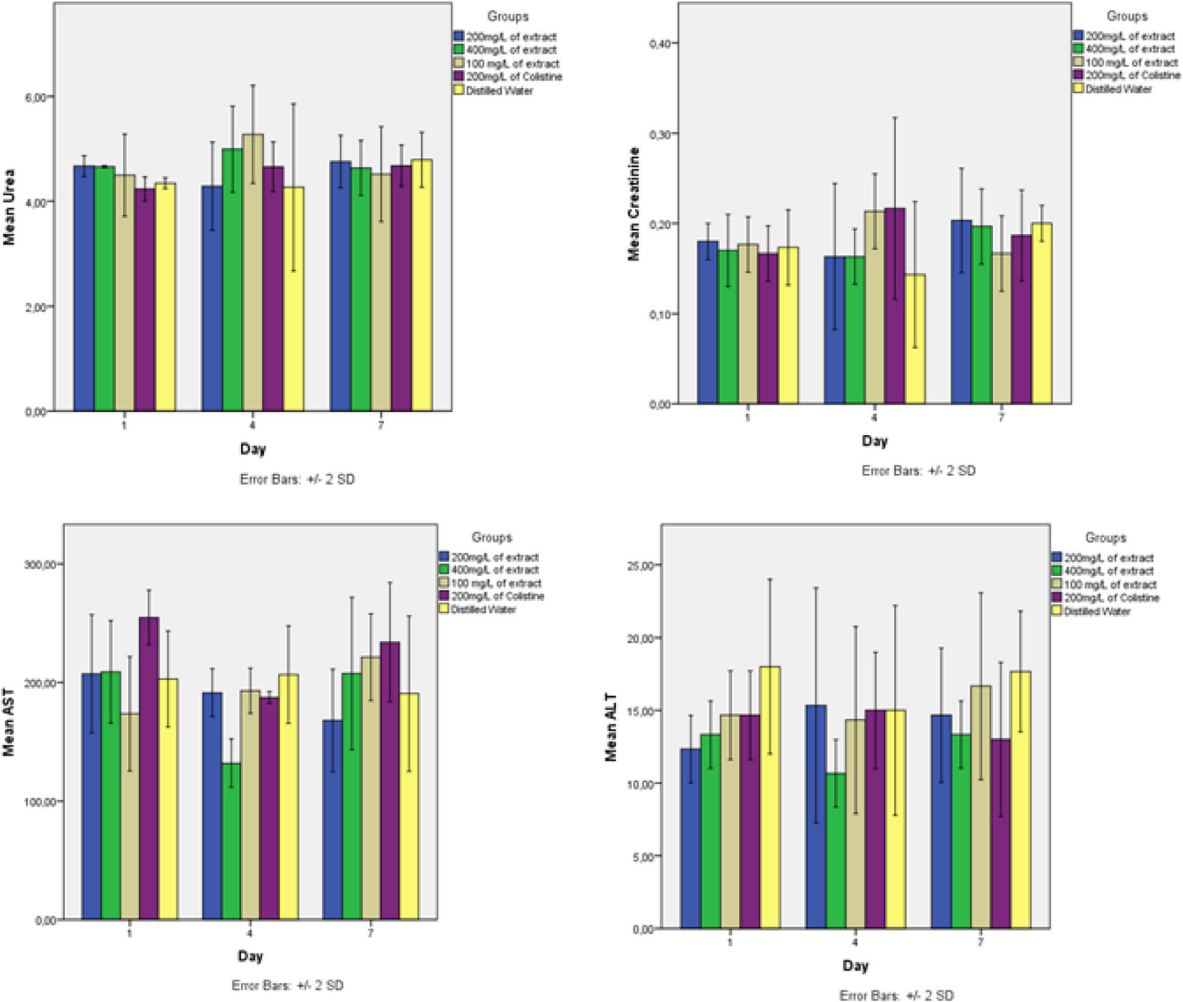
Effect of Leaves aqueous extract of *Uvaria chamae* on Biochemical parameters of Three-week-old chicks

**Fig. 10:**
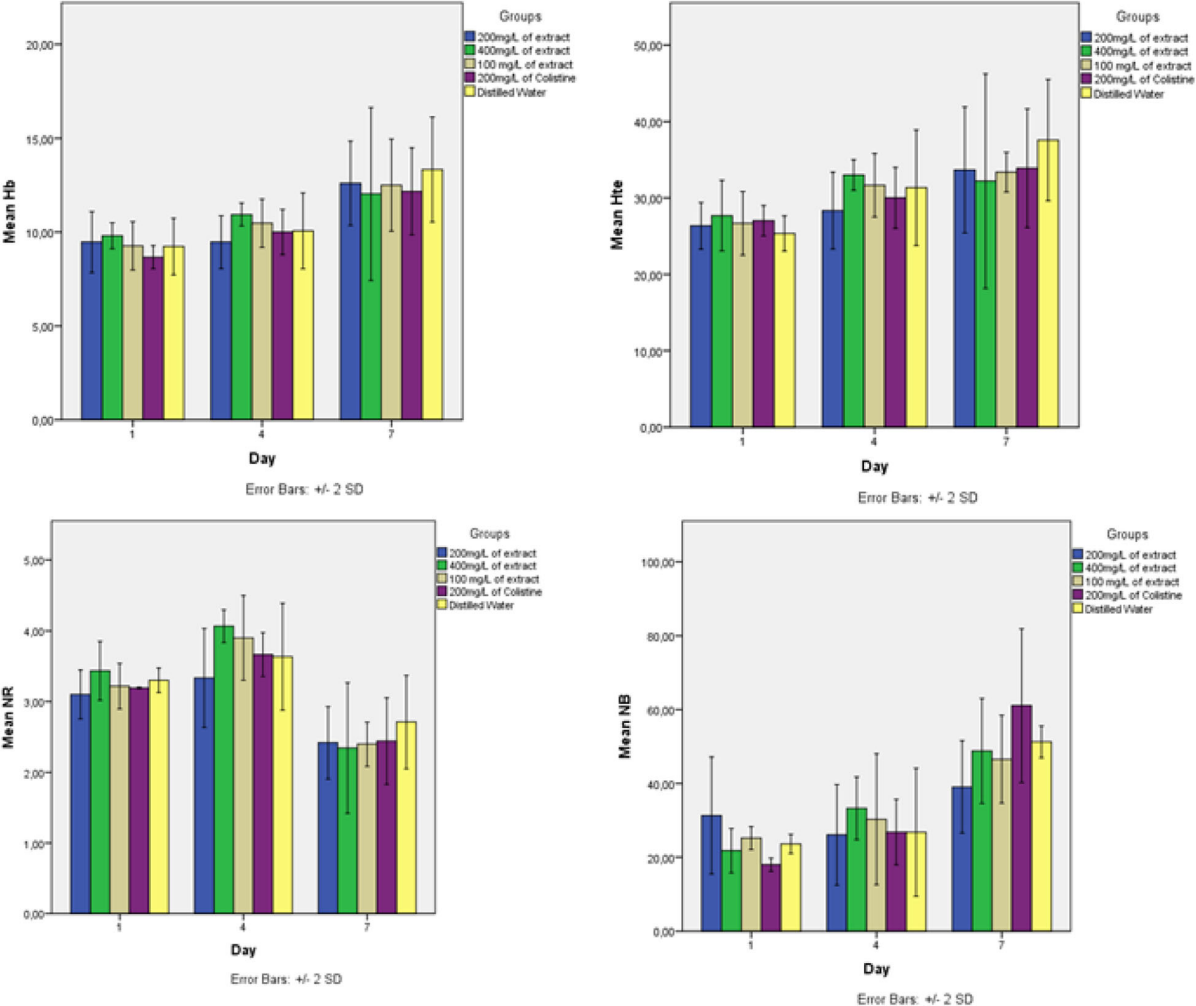
Effect of Leaves aqueous extract of *Uvaria chamae* on Haematological parameters of Three-week-old chicks

Effect of Leaves aqueous extract of *Uvaria chamae* on hematological and biochemical parameters was investigated to evaluate whether as a biologically active substance, this extract did not have a pathological effect on certain biochemical and hematological parameters. The results are shown in Figs. 9 and 10.

Uremia increased insignificantly (*p* ≥ 0.05) at day 4 in chicks which received 400 mg/l (4.66 g/l to 4.99 g/l) and 100 mg/l (4, 49 g/l at 5.27 g/l) of extract. As for those which received 200 mg/l of extract, their uremia increased at day 7 (4.23 g/l to 4.67 g/l). The creatinine concentration at day 4 increased insignificantly in chicks having 100 mg of extract (0.17 g/l to 0.21 g/l) and those which received the antibiotic (0.16 g/l to 0.21 g/l). In chicks which received 200 mg (0.16 g/l to 0.18 g/l) and 400 mg (0.17 g/l to 0.19 g/l) of extract, their creatinine increased at day 7 (*p* ≥ 0.05)). Same observation was done with AST and ALT (no significant variation) (Fig. 9). With hematological parameters, the same observations are made. There was no significant difference in all groups. However, the number of blood cells increased from the 1st to the 4th day and from the 4th to the 7th day (Fig. 10).

## Discussion

### In vitro anti-*Salmonella* activity of *Uvaria chamae*, *Phyllantus amarus* and *Lantana camara*

The aqueous and ethanolic extracts of the leaves and bark of *U. chamae* showed an inhibition of *Salmonella* spp. with the exception of the ethanolic extract of *U. chamae’s* roots. The aqueous extract of *Uvaria chamae* was active on 90% of the virulent *Salmonella* spp. and on *Salmonella* Typhimurium ATCC 14028. These results can be compared to those obtained by Ogueke [17]. This author showed that at a concentration varying between 150 and 250 mg/ml, aqueous and ethanolic extracts of bark and the ethanolic extract of leaves of *Uvaria chamae* inhibited *Salmonella* Typhi.

Aqueous and ethanolic extracts of *Phyllantus amarus* inhibited *Salmonella* spp. with maximal inhibition diameter of 12 mm. The activity of *Phyllantus amarus* extracts on *Salmonella* spp. were reported in 2008. Using agar cup diffusion method, the authors showed that ethanolic extracts of *P. amarus* were active on *Salmonella Typhi* [18]. In our study, only leaves ethanolic extract of *Phyllantus amarus* inhibit *Salmonella Typhimurium* ATCC 14028 (9.33 ± 1.53 mm). The inhibitory power of the extract on the reference strain was greater than those obtained in a previous study. For concentrations ranging from 200 to 1000 μg/ml, the inhibition diameters varied between 7 and 9 mm on *Salmonella* Typhimurium ATCC 6539 [19].

The ethanolic leaves extract of *Lantana camara* had an inhibition diameter of 8 mm on *Salmonella* Typhimurium ATCC 14028. The inhibitory power of extracts of *Lantana camara* on *Salmonella* spp. has already been reported in the literature. Lyumugabe et al. [20] obtained an inhibition diameter of 11 mm on *Salmonella* Typhimurium.

### Experimental infection of three-day-old chicks with *Salmonella* Typhimurium ATCC 14028: preliminary test

To achieve an experimental infection requires that certain experimental conditions be met. The most important are the virulence of the strain used, the choice of the appropriate study model, the choice of the dose and the optimal infective concentration. The choice of chicks as a study model was motivated by two main reasons.

In this study, we choose three-day old chicks for various reasons. One had to choose an age of susceptibility, an age when the animal’s immune system is not mature enough to prevent infection. Such an age guarantees the establishment of the infection. Also it is known that in poultry, the signs of the disease are rarely observed after the first 2 weeks of life [21]. These strategic choices appear to be optimal since, 24 h after the infection, the animals showed signs of salmonellosis, particularly in groups 2 and 3, which received the concentrations of inoculum 2 and 3 respectively. It was observed wet cloaca, diarrhea stool and somnolence. Clinical signs have been associated with the detection of salmonella in feces. The chosen model has therefore made it possible to reproduce the disease. Several studies have focused on the use of chicks at an age of susceptibility to optimize infection. Osman et al. [22] used 1-day-old SPF White Leghorn chicks for inoculation with *Salmonella* Typhimurium. Beal et al. [23] confirmed that *Salmonella* Typhimurium is a non-pathogenic commensal in chickens greater than 3 days of age and can colonize the tract sub-clinically for 8–9 weeks after experimental infection. However, it is possible to use older birds and have interesting results. It all depends on the nature of the study, the virulence of the strain, the concentration and the infective dose. For example, Pande et al. [24] used with success henses of 14 weeks for oral induction with *S.* Typhimurium PT 9. The virulence of *Salmonella* Typhimurium ATCC 14028 was a guarantee because these virulence factors have been characterized by PCR by Deguenon et al. [25]. This study showed that the strain has at least 5 virulence genes: invA, spvR, SpvC, FimA and Stn. Spv genes are responsible for the systemic infection and multidrug resistance in humans and animals [26]. SpvC gene is able to inhibit the activation of macrophages [27]. Presence of fimA gene indicates the presence of fimbriae which is important for *Salmonella* spp. to adhere to epithelial cells [25].

Stn gene is suspected to contribute to enterotoxigenic potency [28]. The presence of all these genes therefore guarantees the pathogenicity of *Salmonella* Typhimurium and its ability to infect chicks.

This virulence explains why salmonellosis symptoms were observed in infected animals. Three infective concentrations were chosen because we had no assurance of sufficient bacterial load to induce salmonellosis in chicks of this age. We had to expand the possibilities. The results showed that only concentrations 2 and 3 could trigger the symptoms of salmonellosis. These symptoms were present until the 10th day of observation in the group 3 chicks while they disappeared between the fifth and the 7th day for the group 2. *Salmonella* spp. were investigated in faecal specimens to support clinical observations. *Salmonella* spp. were detected in chicks of group 2 and 3, 3 days after infection. Chickens infected with concentration 3 of inoculum still host *Salmonella*.

By relating microbiological data to clinical observations, it seems obvious that only the infective 3 concentration was able to keep the 3-day-old chicks sicked for 10 days. This observation was reinforced by the count at day 9. The disappearance of *Salmonella* in group 2 could be explained by a positive reaction of the immune system of the birds.

### In vivo anti-*Salmonella* activity of *Uvaria chamae* using chick model

Twenty-four hours after induction, symptoms were detected in all infected chicks. Diarrheal stools were abundant. The cloacae of the chicks were wet and somnolence was noticed in some chicks. During the 9 days of monitoring, including 7 days of treatment, these symptoms evolved considerably according to the groups. They remained persistent in infected and untreated chicks. It is important to note that in these two lots, diarrhea was slightly reduced at the last days of monitoring. Also the other symptoms such as the appearance of the cloaca became less and less remarkable in certain chicks of the untreated group, even without treatment. This shows a progression of the disease to asymptomatic carriage. As many authors reported, in poultry, salmonellosis is often asymptomatic and symptoms are less and less remarkable after one to 2 weeks of life. But the chicks shed it in their feces. It is for this reason that the experiment was carried out on three-day-old chicks [29–32]. Similarly, this argument undermines the use of symptoms of salmonellosis to judge the efficacy of a therapeutic anti-*salmonella* substance in chicks. This is what led us to carry out the enumeration of *salmonella.*

Leaves aqueous extract of *U. chamae* inhibit *Salmonella* Typhimurium ATCC in chicks at 100, 200 and 400 mg/L but the bacterial load was not canceled. This confirms the in vitro anti-*Salmonella* tests results which showed that this extract has a bacteriostatic effect on *Salmonella* Typhimurium ATCC 14028. On the other hand, at 200 and 400 mg/L, the extract showed a better in vivo activity than Colistin on virulent *Salmonella spp* P19. There is no scientific data on the in vivo activity of extracts of *Uvaria chamae* on *Salmonella spp* using chicks model. But interesting data exist about other natural substances. In a last study, Five-months-old local chickens, free of antibodies against fowl typhoid were used for challenge with *Salmonella* Gallinarum. Administration of extract of *Aloe secundiflora* showed increase in the levels of interleukin 6 (IL-6) [33].

The initial decrease in bacterial load of untreated chickens can be explain by the fact that the animals’ immune system try to control the infection at first. But in the absence of immunity and specific treatment, bacteria continue to multiply, which increases the bacterial load.

## Conclusions

This study demonstrated the in vitro inhibitory potency of aqueous and ethanolic extracts of *Uvaria chamae, Phyllantus amarus and Lantana camara* on *Salmonella spp*. Leaves aqueous extract of *U. chamae* inhibit *Salmonella* Typhimurium ATCC 14028 and *Salmonella* spp. in chicks at 200 and 400 mg/L. The extract showed no toxicity at the concentrations tested. This extract could be enhanced by the development of an improved traditional medicine for the management of non-typhoid salmonellosis.

## Methods

Leaves of *Lantana camara* (Verbenaceae), Leaves and roots of *Uvaria chamae* (Annonaceae), Leaves of *Phyllanthus amarus* Schumach.&.Thonn were collected from the wild. In Benin, no permission was necessary to collect these samples.

Identification was done at the Beninese National Herbarium (University of Abomey Calavi) by Professor Hounnankpon YEDOMONHAN (https://chercheurs.inrab.org/details/173).

Reference numbers in the herbarium are AA6686/HNB for *Phyllantus amarus*, AA6687/HNB for *Uvaria chamae*, AA6688/HNB for *Lantana camara*. This experimental research has been done in compliance with our institution (University of Abomey-Calavi), national and international guidelines. All the plants collected in this study have been replaced by young ones in order to maintain the species survival

Two hundred ten three-day old and 90 three-week old *Isa Brown* male chicks were used for the experimentation. The birds were taken from a commercial hatchery “Terre et Associés”, Abomey-Calavi (Benin). The birds were kept in an enclosure carefully cleaned and disinfected. During the experiment, the animals took water and feed. All experiments were conducted according to the protocol approved by Ethical committee of Research Unit of Applied Microbiology and Pharmacology of natural substances. After the procedure, animals were killed in compliance with the Beninese code for the care and use of animals for scientific purposes. All animal restraint for killing was ethically carried out carefully to avoid fear, distress or pain. In order to limit fear, distress or pain related to restraint, ketamine was administered to animals.

Eleven bacterial strains were used:

- *Salmonella* Typhimurium ATCC 14028 was acquired from Research Unit in Applied Microbiology and Pharmacology of natural substances, University of Abomey-Calavi, Benin.
- Ten multiresistant strains of *Salmonella spp:* they were isolated by Deguenon et al. [25]. The strains were multidrug-resistant to penicillins, first generation cephalosporins and some aminoglycosides.

### Production of aqueous and ethanolic extracts

Method described by Legba et al. [16] were used. After their collection, the organs (leaves and root) were dried at a temperature of 16 °C in the laboratory. Powdering was performed using a Retsch SM 2000/1430/Upm/Smf type mill.

The extraction is performed by macerating fifty grams of dried powder in 500 ml of ethanol or distilled water on a Stuart Bioblock Scientific Fisher stirrer for 72 h. Then, the homogenate was filtered three time with hydrophilic cotton and once with Wattman paper. The filtrate obtained was dried at 40 °C for ethanolic extract and 50 °C for aqueous extract in the Pasteur oven.

### In vitro anti-*Salmonella* assessment of aqueous and ethanolic extract of *Uvaria chamae*, *Phyllantus amarus* and *Lantana camara*

*Salmonella* Typhimurium 14,028 and the ten multiresistant *Salmonella* spp. were used. Inoculum was obtained by emulsifying a 24-h pure colony of each strain from the Mueller Hinton (MH) medium in physiological water (5 ml). A turbidity of 0.5 Mc Farland is obtained [16].

Using swab, each inoculum was seeded on Petri dishes containing MH agar. Sterile Pasteur pipette tip is then used to hollow out 6 mm Wells. 50 μl of each extract were taken and deposited in the wells. Negative control was constituted by well which contain sterile distilled water. After 1 h pre-diffusion at room temperature, the petri dish was incubated at 37 °C. After 24 h of incubation, the dish was examined and the zones of inhibition were measured [16].

Method used by Legba et al. [33] were used for determination of Minimal Inhibitory Concentration (MIC) and Minimal Bactericidal Concentration (MBC). Each extract were prepared at 200 mg/ml. 100 μl of the stock solution were added to 100 μl of MH Broth. A series of two-fold dilution was made from well to well, then 100 μl of different inoculum were added. Positive and negative controls were constituted respectively by adding 100 μl of MH broth to 100 μl of inoculum and 100 μl of MH broth to 100 μl of the extract. The plates were incubated at 37 °C in bacteriological oven. The MIC was estimated after 24 h of incubation using Tetrazolium. For MBC determination, the content of each well was cultured on MH Agar and incubated at 37 °C for 24 h. The lowest concentration of extract for which no colony of bacteria is observed is MBC. The formula CMB/CMI was used to calculate the antibiotic potency (a.p) of each extract.

### Experimental infection of three-day-old *Isa Brown* male chicks with *Salmonella* Typhimurium ATCC 14028

A preliminary microbiological examination of the cloaca of the chicks helped to check if chicks are exempt from *Salmonella spp*. A cloaca swab was performed on all chicks and *Salmonella spp* were searched according to the method described by Deguenon et al. [25].

*Salmonella* inoculums were prepared from a pure isolate of the bacterial strain in distilled water at three selected concentrations: 3 × 10^8^ CFU (Concentration 1), 6 × 10^8^ CFU (Concentration 2), 9 × 10^8^ CFU (Concentration 3).

- Groups 1 (*n* = 3): 2 ml of Inoculum concentration 1
- Groups 2 (*n* = 3): 2 ml of Inoculum concentration 2
- Groups 3 (*n* = 3): 2 ml of Inoculum concentration 3
- Groups 4 (*n* = 3): 2 ml of Distilled water

Oral inoculation was performed using 20-gauge feeding needle and disposal syringe. To confirm the viability of the strain used for inoculation, a sample of each inoculum (about 100 μl) was taken before and after oral administration for culture at 37 °C for 24–45 h.

The birds were observed for 10 days and the symptoms of salmonellosis were recorded. On days 3, 6, and 9, the feces from each lots of chicks were collected. Salmonella was sought using method described par Deguenon et al. [25]. Five (5) grams of fecal samples were transferred immediately following collection in 45 ml of buffered peptone water. Samples were homogenized in the broth either by vortex mixer and then incubated for 18–24 h at 37 °C. 1 ml of pre-enrichment was then inoculated in 9 ml of selenite cystine broth for 24 h. Isolation was done on petri-dishes containing Xylose Lysine Decarboxylase (XLD) and incubated for 24 h. API 20E Gallery was used for positive identification of all suspicious colonies from fecal material. *Salmonella* counts were performed on Day 9 samples to assess bacterial load. The method described by Pande et al. [24].

### In vivo anti-*Salmonella* assessment of leaves aqueous extract of *Uvaria chamae*

*Salmonella* Typhimurium 14,028 and *Salmonella* spp. (P19) were used for this step. After inoculation, chicks were treated with leaves aqueous extract of *Uvaria chamae* and colistin.

For each strain**,** 90 three-day-old chicks were randomly assorted into six groups:

- Group 1: non-infected and non-treated (G1, *n* = 18)
- Group 2: infected and untreated (G2, *n* = 18)
- Group 3: infected and treated with 200 mg/l of Colistin (G3, *n* = 18)
- Group 4: infected and treated with 100 mg/l of *Uvaria chamae* leaves aqueous extract (G4, *n* = 18)
- Group 5: infected and treated with 200 mg/l of *Uvaria chamae* leaves aqueous extract (G5, *n* = 18)
- Group 6: infected and treated with 400 mg/l of *Uvaria chamae* leaves aqueous extract (G6, *n* = 18).

Preliminary examination and inoculation were performed as previously but a single concentration of inoculum were used: 9.0 10^8^ UFC/ml. The birds were observed for 9 days and the symptoms of salmonellosis were recorded. From the third day after infection, the chicks are subjected to oral treatment with the aqueous leaf extract of *Uvaria chamae* and colistin as reference antibiotic. The treatment was done for 7 days. On days 3, 6 and 9 after infection, faeces from each group were collected and *Salmonella* counts were performed [34].

### Effects of leaves aqueous extract of *Uvaria chamae* on biochemical and hematological parameters

In order to evaluate the toxicity of *Uvaria chamae* leaves aqueous extracts for chicks, the different concentrations of extracts tested and Colistin were administered for 7 days to three-week-old chicks.

Ninety three-week-old Isa Brown male chicks were divided in five groups:

- Group 1: oral administration of 100 mg/L of Leaves aqueous extract of *U. chamae* (G1, n = 18)
- Group 2: oral administration of 200 mg/L of Leaves aqueous extract of *U.chamae* (G2, n = 18)
- Group 3: oral administration of 400 mg/L of Leaves aqueous extract of *U.chamae* (G3, n = 18)
- Group 4: oral administration of Colistin (G4, n = 18)
- Group 5: water

The hematological (white cell Number (NB); Blood red Cell (NR), Hemoglobin (Hb), Hematocrit (Hte) and biochemical (Uremia, creatinine, AST and ALT) data were recorded on Days 0, 4 and 7.

### Euthanasia

For euthanasia, we used a technique from the “Guidelines for the Euthanasia of Animals, and the 2016 Canadian Code of Practice for the care and handling of Hatching Eggs, Breeders, Chickens and Turkeys” [35]. Since the animals are chicks, the most recommended method is carbon dioxide (CO_2_) absorption. The chicks were exposed to carbon dioxide (CO_2_) in a CO_2_ chamber. A heater coil has been added to the supply line in order to prevent interruption of gas due to freezing. Confirmation of euthanasia have been done on every chick, using lack of nictitating membrane reflex. Dilated and fixed pupil served as a sign of unconsciousness and/or death [35]. When removed from the chamber, a secondary method of euthanasia before birds regain consciousness if the birds are not dead. Then, 80 mg per weight kilogram of Sodium thiopental (intravenous injection) were administered to them.

### Statistical analysis

Microbiological data were analyzed using ANOVA test with Graph Pad Prism 7.0 Software. Hematological and Biochemical data were analyzed by the SPSS 17.0 Software.

## Data Availability

All data generated or analyzed during this study is included in this published article and supplementary information files.

## Abbreviations

a.p: Antibiotic potency
ALT: Alanine aminotransferase
ANOVA: Analysis of Variance
API: Analytical Profile Index
AST: Aspartate transaminase
ATCC: American Type Culture Collection
CFU: Colony forming unit
G: Group
HNB: National Herbarium of Benin
MBC: Minimum Bactericidal Concentration
MH: Mueller Hinton
MIC: Minimum Inhibitory Concentration
P9, P70, P16, P14, P15, P19, P362, P368, P17, P29: *Salmonella* samples identification code
PCR: Polymerase chain reaction
SPF: Specific-Pathogen-Free
SPSS: Statistical Package for the Social Sciences
ST: *Salmonella* Typhimurium
TWAS: The World Academy of Sciences
UNESCO: United Nations Educational, Scientific and Cultural Organization
XLD: Xylose Lysine Decarboxylase
